# Epigenetic remodeling of the immune landscape in cancer: therapeutic hurdles and opportunities

**DOI:** 10.1186/s12929-022-00893-0

**Published:** 2023-01-10

**Authors:** Feng-Ming Tien, Hsuan-Hsuan Lu, Shu-Yung Lin, Hsing-Chen Tsai

**Affiliations:** 1 Department of Internal Medicine, National Taiwan University Hospital, Taipei, 100225 Taiwan; 2 Graduate Institute of Clinical Medicine, College of Medicine, National Taiwan University, Taipei, 100233 Taiwan; 3 Center for Frontier Medicine, National Taiwan University Hospital, Taipei, 100225 Taiwan; 4 Graduate Institute of Toxicology, College of Medicine, National Taiwan University, No. 1 Jen Ai Road Section 1, Rm542, Taipei, 100233 Taiwan; 5 Department of Medical Research, National Taiwan University Hospital, Taipei, 100225 Taiwan

**Keywords:** Tumor immune microenvironment, Epigenetic therapy, Immunotherapy, DNA methylation, Histone modifications, Chromatin accessibility, Innate immunity, Adaptive immunity

## Abstract

The tumor immune microenvironment represents a sophisticated ecosystem where various immune cell subtypes communicate with cancer cells and stromal cells. The dynamic cellular composition and functional characteristics of the immune landscape along the trajectory of cancer development greatly impact the therapeutic efficacy and clinical outcome in patients receiving systemic antitumor therapy. Mounting evidence has suggested that epigenetic mechanisms are the underpinning of many aspects of antitumor immunity and facilitate immune state transitions during differentiation, activation, inhibition, or dysfunction. Thus, targeting epigenetic modifiers to remodel the immune microenvironment holds great potential as an integral part of anticancer regimens. In this review, we summarize the epigenetic profiles and key epigenetic modifiers in individual immune cell types that define the functional coordinates of tumor permissive and non-permissive immune landscapes. We discuss the immunomodulatory roles of current and prospective epigenetic therapeutic agents, which may open new opportunities in enhancing cancer immunotherapy or overcoming existing therapeutic challenges in the management of cancer.

## Background

Complex cellular dynamics and functional plasticity shape the landscape of the tumor immune microenvironment (TIME) and determine the fate of tumor progression. Targeting the cellular components and their interactions within the TIME holds promise as anti-cancer therapeutic strategies. Among many molecular and signaling networks that regulate the temporal and spatial remodeling of the TIME, epigenetic regulatory processes play a key role in orchestrating the interplay between immune-immune and immune-tumor cells. Epigenetic mechanisms comprise a multi-layer regulatory system that modulates transcriptomic patterns of mammalian cells in a coordinated manner without changes in primary DNA sequences. These control mechanisms include chemical modifications of DNA and histones, as well as the accessibility of chromatins, among others [1]. The entire regulatory scheme appears to be dynamic and sophisticated, often leading to a functional switch in response to various environmental cues during cancer progression. Rigorous research efforts have focused on uncovering the epigenetic players that mediate differentiation and activation of individual immune cell types, which have been summarized elsewhere [2–8]. In this review, we will highlight epigenetic regulators that mediate the protumor or antitumor function of the TIME from the therapeutic perspective, particularly in the cell types directly involved in tumor lytic activities, such as natural killer (NK) cells, T cells, and macrophages. We will examine how epigenetic processes affect the individual cellular components of the TIME as well as their interactions with tumor cells via immune synapses. We will also enlist several U.S. FDA-approved epigenetic therapeutic agents and describe their roles in modulating the immune landscape in cancer. Deciphering these epigenetic-immune networks will pave the way to the development of pharmacological strategies for remodeling the TIME, thereby overcoming drug resistance or enhancing treatment responses for better therapeutic outcomes in cancer patients.

## Major epigenetic mechanisms in TIME

The TIME is composed of various immune cells with diverse functions. These immune cells can distribute in clusters, interspersed within tumor cells or surrounding tumor margins in response to tumor-intrinsic or extrinsic signals. Analysis of cell composition, spatial distribution, functional and transcriptional analysis provides an archetypal classification of the TIME, which dictates the vulnerability of the tumors to different immunotherapeutic strategies [9–11]. Compelling evidence has linked these functional classifications of TIME to treatment responses and clinical outcomes. Moreover, a detailed analysis of tumor-infiltrating immune cell subtypes provides additional indicators for patient prognosis [12–14]. For instance, higher levels of estimated effector T cell fractions are generally correlated with better survival, while increasing levels of myeloid populations are primarily associated with poorer survival [13, 15]. On the other hand, tumor-associated M2 macrophages predict worse outcomes as opposed to pro-inflammatory M1 macrophages [16, 17]. These prognostic landscapes of individual immune cell types within the TIME highlight the importance of understanding the fundamental regulatory mechanisms that delineate the complexity of cell composition, mediate functional plasticity and tip the balance between anti-tumor and pro-tumor immunities to affect clinical outcomes.

Distinct epigenetic features of lymphoid and myeloid lineages at different differentiation stages and diverse functional states have been demonstrated in various studies [18–20]. Key epigenetic mechanisms in regulating immune-functional states include DNA methylation, histone modifications, and chromatin accessibility, among others. DNA methylation is a biological process in which a methyl group (–CH3) from S-adenosylmethionine (SAM) is added to the 5’ position of the pyrimidine ring of cytosines. This chemical reaction is catalyzed by a group of enzymes named DNA methyltransferases (DNMTs). By contrast, TETs (Ten-eleven translocation enzymes), also known as methylcytosine dioxygenases, facilitate passive and active DNA demethylation by catalyzing 5-methylcytosine oxidation to produce 5-hydroxymethylcytosine (5hmC) and other methylcytosine metabolites [21]. DNA methylation can occur at various regulatory elements throughout the genome. In the gene promoters, DNA methylation usually correlates with transcriptional gene silencing. In other regions, it can modulate enhancer activity, gene activation, and mRNA splicing [22]. Abundant evidence has indicated that DNMTs play a significant role in modulating functional states of diverse immune cell subtypes in the innate and adaptive systems in both methylation-dependent and -independent manners. On the other hand, TET proteins can fine-tune DNA methylation patterns and shape immune responses via modulating immune-related gene expressions [23]. Therefore, genetic or pharmacological inhibition of DNMTs or TETs holds great promise in remodeling the TIME via cell type-specific reprogramming of immune cells and their interaction with cancer cells.

Histone modifications refer to covalent chemical modifications on the histone N-terminal tails (e.g., acetylation, methylation, and phosphorylation) that mediate gene activities and cellular states [24, 25]. Addition, recognition, and removal of histone modifications are carried out by different classes of histone modifying enzymes—writers [e.g., histone acetyltransferases (HATs); histone methyltransferases(HMTs), etc.], readers (e.g., bromodomains, chromodomains, etc.), and erasers (e.g., histone deacetylases(HDACs), lysine demethylases(KDMs), etc.) [26–28]. Notably, the immunomodulatory activities of many histone modifying enzymes have been revealed, including histone methyltransferases—enhancer of zeste homolog 2 (EZH2) [29, 30], DOT1 like histone lysine methyltransferase (DOT1L) [31], SET domain containing 4 (SETD4) [32], histone demethylase—lysine-specific histone demethylase 1A (KDM1A)[33], lysine demethylase 6B (KDM6B) [34, 35], histone deacetylases—sirtuin 6 (SIRT6) [36], HDACs) [37–39], readers of acetyl histones—bromodomain-containing protein 4 (BRD4) [40], and others. These histone-modifying enzymes often partner with other transcriptional activators or repressors in a protein complex to exert gene regulatory activities and may sometimes play opposite roles in shaping antitumor immunity in a context-dependent manner. Numerous drugs that target these histone-modifying enzymes are under active development as therapeutic agents for cancer and other diseases. A few have been FDA-approved for the management of hematological malignancies, such as inhibitors of HDAC (i.e., panobinostat, romidepsin, belinostat, vorinostat) [41] and EZH2 (i.e., tazemetostat) [42, 43]. While the anti-tumor activities of these drugs have been well demonstrated, how the drugs modulate the TIME and individual immune cell subtypes to affect therapeutic responses and prognostic outcomes is still awaiting in-depth investigation.

Chromatin accessibility, another epigenetic regulatory mechanism, refers to the level of physical access to chromatinized DNA. Accessible chromatin allows for the binding of transcriptional mediators to regulatory DNA elements (e.g., enhancers and promoters) and can infer transcriptional patterns and gene activities [44]. As technologies for genome-wide chromatin accessibility profiling rapidly advance [45–49], scientists are gaining significant insights into the complexity of regulatory circuits in rare cell populations or at single-cell resolution within the heterogenous immune microenvironment. The information on genome-wide chromatin accessibility provides a valuable tool to characterize cell identity and functional states that may or may not be clearly delineated by genomics and transcriptomes. Chromatin remodeling is an ATP-dependent process carried out by various chromatin remodeling complexes, including SWItch/sucrose non-fermentable (SWI/SNF), imitation switch (ISWI), Mi-2/nucleosome remodeling and deacetylase (NuRD), and INO80 complexes, etc. Inhibitors or activators of these chromatin remodelers are being actively explored for their potential clinical benefits, although our knowledge of the precise mechanistic link between therapeutic reprogramming of chromatin accessibility and functional switch of immune states remains limited.

Along the trajectory of cancer initiation and progression, it is believed that these central epigenetic mechanisms orchestrate the functional dynamics of our immune system. According to the BLUEPRINT Epigenome Project, the epigenomic analyses of 112 samples from the human immune system revealed that global methylation levels decline progressively along the differentiation spectrum in both T and B lymphocytes. Moreover, lineage-specific epigenetic patterns, including DNA methylation, histone modifications, and chromatin accessibilities, differentially defined the innate and adaptive immune systems [20, 50, 51]. Particularly, both immune systems are subject to epigenetic control to mount anti-tumor responses or exert immunosuppressive effects in the tumor microenvironment. The dominance of pro-tumor immunological effects over anti-tumor responses will eventually create a permissive microenvironment to allow for tumor development and progression. For example, in the innate immunity system, whether macrophage has a pro-tumor effect or an anti-tumor effect is governed by epigenetic control. Two epigenetic proteins, DNMTs and TETs, govern macrophage polarization in opposite directions: DNMTs promote M1 macrophage polarization, while TET2 promotes M2 macrophage polarization [2, 52]. Similarly, the dual effect of epigenetic control can be observed in the adaptive immune system. EZH2 increases anti-tumor immunity of CD8+ T cells while mediates the immunosuppressive function of Treg cells [53, 54].” These findings will be elaborated further in the following sections.

## Epigenetic regulation of tumor-associated immune responses

During tumor progression, malignant cells display varying degrees of immunogenicity and elicit responses from the innate and adaptive immune systems. These tumor-associated immune responses are the collective result of cell type-specific epigenetic regulation in immune cells at the encounter of cancer cells (Table 1). Studies on epigenetic mechanisms that orchestrate the cellular components of the TIME may open the possibility of therapeutic intervention to convert a tumor-permissive niche into a non-permissive one and to enhance the efficacy of cancer immunotherapy in the clinical setting.

**Table 1:** Phenotypic and functional effects of epigenetic modifiers in key immune cell types

Cell type	Epigenetic modifier		Locus	Effects	References
NK cells	DNMTs	DNA methyltransferase	DNA methylation	• Enhance IFN-γ expression • Regulate KIR expression	Chan et al. [63] Luetke-Eversloh et al [66] Sohlberg et al. [69]
	EZH2	Histone methyltransferase	H3K27me3	• Suppress IL-15R (CD122) and NKG2D expressions	Yin et al. [29] Bugide et al. [30]
	ASH1L	Histone methyltransferase	H3K36me3	• Positively regulate NK activation	Li et al. [62]
	JARID2	Histone methyltransferase	H3K27me3	• Positively regulate NK activation	Li et al. [62]
	KDM6B	Histone demethylase	H3K27me2/3	• Positively regulate NK activation	Li et al. [62]
	UTY	Histone demethylase	H3K27me	• Positively regulate NK activation	Li et al. [62]
Macrophages	DNMTs	DNA methyltransferase	DNA methylation at promoters of *Klf4*, *Socs1*, *Pparg*	• Promote M1 polarization	Cheng et al. [75] Yang et al. [76] Niu et al. [2]
	TET2	Methylcytosine dioxygenases	DNA methylation at promoters of immunosuppressive genes	• Promote M2 polarization	Pan et al. [77] Lio et al. [23]
	EHMT2	Histone methyltransferase	H3K9me3	• Suppress M1 polarization	Wang et al. [82]
	ASH1L	Histone methyltransferase	H3K4me3	• Suppress IL-6 and TNFα production	Xia et al. [197]
	SETD4	Histone methyltransferase	H3K4me1, H3K4me2	• Induce macrophage activation • Increase IL-6 and TNFα expression	Zhong et al. [32]
	SETD7	Histone methyltransferase	H3K4me1	• Promote M1 polarization, • Increased *S100A9* and *S100A12* expressions	Mossel et al. [83]
	SMYD2	Histone methyltransferase	H3K36me2	• Negative regulation of macrophage activation	Xu et al. [78]
	SMYD3	Histone methyltransferase	H3K4me3	• Promote M1 polarization, Increased *S100A9* and *S100A12* expressions	Mossel et al. [83]
	SMYD5	Histone methyltransferase	H4K20me3	• Negative regulation of macrophage activation	Stender et al. [79]
	KDM6B	Histone demethylase	H3K27me	• Promote M2 polarization	Ishii et al. [80] Satoh et al. [81] Yıldırım-Buharalıoğlu et al. [198] Xun et al. [34] Raines et al. [35]
	HDAC11	Histone deacetylase	AcH3	• Inhibit M2 polarization and activation	Hu et al. [199] Shinohara et al. [38]
	KAT2A/B	Histone acetyltransferase	H3K9ac	• Promote M2 activation • Increase IL-6 expression	Hu et al. [199]
	EP300	Histone acetyltransferase	Acetyl Histone H3	• Promote M2 activation • Increase IL-6 expression	Wang et al. [84]
	PRMT1	Arginine methyltransferase	Arginine methylation of c-Myc	• Promote M2 activation	Tikhanovich et al. [85]
CD8 + T cells	DNMT3A	DNA methyltransferase	DNA methylation at promoters of *Eomes*, *Tbx21* and *Tcf7 and* promoters of naive-associated genes	• Promote tumor-specific T cell dysfunction • Establish de novo DNA methylation program for T cell exhaustion • Repress naive-associated genes in effector CD8+ T cells	Schietinger et al [97] Ghoneim et al. [96] Youngblood et al [200]
	TET2	Methylcytosine dioxygenases	DNA methylation	• Regulate CD8+ T cell memory differentiation • Promote terminal exhaustion of T cells	Carty et al. [201] Jordan et al. [98]
	EZH2	Histone methyltransferase	H3K27me3	• Promote SLEC and effector T cell differentiation • Anti-tumor immunity	Karantanos et al. [53] Schietinger et al. [97] Zhao et al. [202] Gray et al. [87]
	EHMT2	Histone methyltransferase	H3K9me3	• Suppress the development of memory precursor CD8^+^ T cells • Repress *Il2ra* and *Cd27*	Shin et al. [89]
	BMI1	Epigenetic repressor, Component of the polycomb group complex 1 (PRC1)	Targets of PRC1 complexes	• Promote T cell activation and expansion	Heffner et al. [203] Henning et al. [4]
	BRD4	Bromodomain-containing protein	Acetylated lysines	• Promote T cell differentiation into an effector memory phenotype • Enhance T cell persistence and antitumor effects	Kagoya et al. [40]
	HDAC2	Histone deacetylase	Acetylated histones	• Suppress the development of memory precursor CD8^+^ T cells • Repress *Il2ra* and *Cd27*	Shin et al. [89]
	KDM1A (LSD1)	Histone demethylase	H3K4me, H3K9me	• Promote terminal differentiation in exhausted CD8 + T cells	Liu et al. [33]
	SIRT1	NAD^+^-dependent deacetylase	N/A	• Inhibit effector T cell differentiation	Kuroda et al. [204]
	TOX	Transcription factor/Chromatin modifier	Binds DNA via the HMG-box motif	• Promote T cell persistence • Drive CD8+ T cell exhaustion	Khan et al. [101] Alfei et al. [103] Scott et al. [102] Yao et al. [205] Seo et al. [206] Wang et al. [207]
Treg	HDAC	Histone deacetylase	FOXP3	• Decreased binding of FOXP3 to chromatin • Destabilize FOXP3+ Treg cells	Samanta et al. [39]
	EZH2	Histone methyltransferase	H3K27me3	• Mediate immunosuppressive functions in tumor-infiltrating Tregs	Wang et al. [113] Goswami et al. [54]
Dendritic cells	TETs	Methylcytosine dioxygenases	DNA methylation	• Modulate dendritic cell activation	Li et al. [208]
	EZH2	Histone methyltransferase	H3K27me	• Promote dendritic cell activation	Li et al. [124]
	DOT1L	Histone methyltransferase	H3K79me2	• Inhibit maturation of BMDCs in cancer	Zhou et al. [31]
	HDAC	Histone deacetylase	Acetylated histones	• Promote maturation of DCs from monocyte	Nencioni et al. [122]
	SIRT6	NAD^+^-dependent deacetylase	H3K9ac	• Promote dendritic cell migration	Ferrara et al. [36]
MDSC	Class I HDACs	Class I histone deacetylases	Acetylated histones	• Promote immune suppressive activity of G-MDSC	Hashimoto et al. [36]
	HDAC2	Class I histone deacetylase	Acetylated histones	• Promote phenotypic switch from M-MDSC to G-MDSC	Youn et al. [37]
	HDAC6	Class IIb histone deacetylase	Acetylated histones	• Promote immune suppressive activity of M-MDSC	Hashimoto et al. [36]
	HDAC11	Class VI histone deacetylase	Acetylated histones	• Negatively regulate MDSC expansion and function	Sahakian et al. [128]

*NK* natural killer, *Treg* regulatory T cells, *MDSC* myeloid-derived suppressor cells

### Epigenetic regulation of the innate immune response—NK cells

Natural killer (NK) cells are the major cellular component of the innate immune response and serve as the first line of defense against transformed and viral-infected cells. NK cells, derived from common lymphoid precursors, are capable of mediating tumor lysis through major histocompatibility complex (MHC)-independent mechanisms [55, 56]. Abundant tumor infiltration of NK cells has been associated with improved patient outcomes as well as favorable responses to immune checkpoint inhibitor therapy [57–59]. In addition, circulating NK cells have been demonstrated to limit the metastatic spread of cancer cells [60, 61]. The activity of NK cells is triggered by engaging activating (e.g., natural killer group 2D (NKG2D), DNAX accessory molecule-1 (DNAM1), natural killer protein 30 (NKp30), natural cytotoxicity triggering receptor 1(NCR1), etc.) or inhibitory receptors (e.g., natural killer group 2A (NKG2A), killer cell immunoglobulin-like receptor, two Ig domains and long cytoplasmic tail 1-3 (KIR2DL1-3), T cell immunoreceptor with Ig and ITIM domains (TIGIT), etc.) on the NK cell surface by specific ligands expressed on tumor cells. In particular, the activating receptor, NKG2D, recognizes various ligands (e.g., MHC class I polypeptide-related sequence A (MICA), MHC class I polypeptide-related sequence B (MICB) and UL16 binding proteins (ULBPs) 1–6) on cancer cells and is critical for tumor lytic abilities of NK cells. Notably, the anti-tumor responses of NK cells can be multifold. Upon activation, NK cells may exert anti-cancer effector functions via direct lysis of cancer cells or indirect cytotoxic responses by secreting cognate ligands [e.g., tumor necrosis factor-related apoptosis-inducing ligand (TRAIL)] for death receptors or pro-inflammatory cytokines [e.g., interferon-gamma (IFN-γ)].

**Fig. 1:**
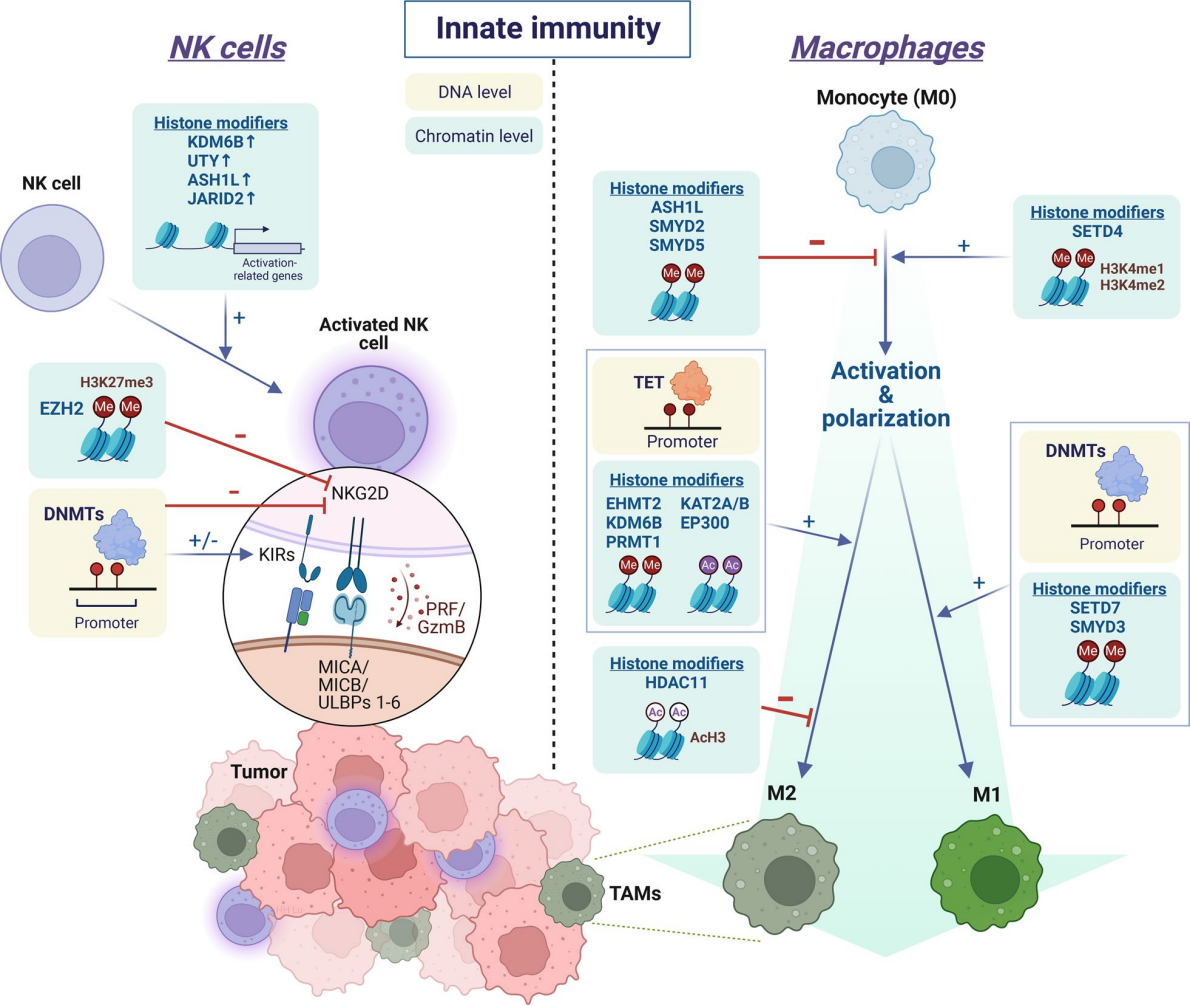
Epigenetic regulation of innate immune cells in the tumor immune microenvironment. In natural killer (NK) cells, histone methyltransferases (ASH1L and JARID2) and histone demethylases (KDM6B and UTY) promote NK activation via upregulating activation-related gene expressions. By contrast, EZH2 and DNMTs suppress the expression of activating NK receptors, such as NKG2D. In macrophages, activation of monocyte (M0) is positively regulated by SETD4 and negatively regulated by ASH1L, SMYD2, and SMYD5, respectively. Epigenetic modifiers are also involved in the polarization process of macrophages. SETD7, SMYD3 and DNMTs promote M1 polarization, whereas EHMT2, KDM6B, PRMT1, KAT2A/B, EP300 and TET promote M2 polarization. Tumor-associated macrophages (TAMs) often exhibit an M2-like phenotype. H3K4me1: mono-methylation at the 4th lysine residue of the histone H3. H3K4me2: di-methylation of lysine 4 on histone H3. H3K27me3: tri-methylation of lysine 27 on histone H3. “Me” in red circle: methyl group. “Ac” in purple circle: acetyl group. AcH3: histone H3 acetylation. KIRs: killer Ig-like receptors. PRF: perforin. GzmB: granzyme B. This figure was created on BioRender.com

The involvement of epigenetic modifiers in the activation of NK cells has been indicated by marked transcriptomic alterations of multiple histone demethylases (i.e., lysine demethylase 6B (KDM6B), ubiquitously transcribed tetratricopeptide repeat containing, y-linked (UTY)) and histone methyltransferases [i.e., ASH1 like histone lysine methyltransferase (ASH1L), and jumonji and AT-rich interaction domain containing 2 (JARID2)] [62]. (Fig. 1) In addition, many studies demonstrated that DNA methylation plays a significant role in regulating the expression of various NK cell receptors, including killer Ig-like receptors (KIRs), which gradually became demethylated and expressed as the chromatin structure opened during differentiation and maturation of NK cells [63–65]. Furthermore, effector cytokines such as IFN-γ are also regulated by DNA methylation in NK cells [66]. Notably, the immunomodulatory effects of DNMT inhibitors (i.e., decitabine, azacitidine), also known as DNA hypomethylating agents, on NK cells remain inconclusive. While several studies suggest that treating NK cells with DNA hypomethylating agents can suppress NK-mediated cancer killing by upregulation of inhibitory KIRs and reduction of granzyme B and perforin release [67, 68], other studies indicate otherwise [69, 70]. Thus, the roles of DNA methylation in mediating NK cell function are probably context- or tissue-dependent.

In addition to DNA methylation, the epigenetic machinery on histone modifications, such as HATs, HDACs, HMTs, and demethylases, also involves in the activation and anti-tumor immunity of NK cells [62]. For instance, histone acetylation, which is often associated with active gene transcription, may promote the expression of IFN-γ and NKG2D in NK cells. Fernandez-Sanchez et al. demonstrated that expression of NKG2D on NK cells is often accompanied by high levels of histone H3 lysine 9 acetylation (H3K9ac). Treatment with histone acetyltransferase inhibitors downregulates NKG2D expression leading to a reduction in NKG2D-mediated cytotoxicity [71]. Perplexedly, the effect of different HDAC inhibitors (HDACi) on the functional phenotypes of NK cells may differ. Non-selective HDACis such as trichostatin A (TSA), valproic acid (VA), and sodium butyrate impair NK cell-mediated cytotoxicity and downregulate the activating receptor, NKG2D [72, 73], whereas a selective class I HDACi, entinostat, was reported to induce NKG2D expression and NK activation [74].

Another important histone modifier that mediates lineage commitment and functional differentiation of NK cells is EZH2, a histone methyltransferase that catalyzes the repressive modification H3K27me3 and often associates with transcriptional inactivation. Genetic disruption or pharmacological inhibition of EZH2 enhances NK cells’ cytotoxicity against cancer cells through induction of interleukin 15 receptor, beta (IL-15Rβ), and NKG2D expressions [29]. These findings support the therapeutic potential for epigenetic intervention on lineage specification and functional regulation of NK cells (Fig. 1).

### Epigenetic regulation of the innate immune response—macrophages

Macrophages are an immune cell type that belongs to the innate immune system and can exert both immunological (i.e., foreign substance clearance, antigen presentation, and cell–cell communications) and non-immunological functions (i.e., wound healing, tissue remodeling, regeneration, etc.) in a tissue-specific manner [2]. Abundant evidence has shown that macrophages are heterogeneous cell populations subject to diverse epigenetic regulations leading to phenotypic plasticities, such as M1 or M2 polarization. M1 macrophages secrete pro-inflammatory signals (e.g., IFN-γ, interleukin 1 (IL-1), interleukin 6 (IL-6), tumor necrosis factor (TNF), etc.) and are a key component of host defense against pathogens, whereas M2 macrophages produce anti-inflammatory signals (e.g., interleukin 4 (IL-4), interleukin 10 (IL-10), transforming growth factor beta (TGF-β), etc.) to prevent inflammation responses. Tumor-associated macrophages (TAMs) refer to those macrophages that infiltrate the TIME, which often adopt an M2-like phenotype characterized by their pro-tumor function such as producing immunosuppressive cytokines, reducing tumor antigen presentation, promoting tumor invasion/metastasis, and enhancing therapeutic resistance.

The differentiation, activation, and polarization states of macrophages are mediated by several epigenetic mechanisms (Fig. 1). Emerging evidence has suggested that DNA methylation machinery, including DNMTs, and TET methylcytosine dioxygenases, plays a significant role in macrophage polarization. While DNMTs tend to promote M1 polarization [75, 76], TET proteins are associated with an M2 phenotype [23]. Depletion of Tet2 in tumor-associated macrophages reduces the immunosuppressive function and inhibits melanoma growth in vivo [77]. Therefore, the opposite effect of DNMTs and TETs on DNA methylation is tuning the balance between the M1 and M2 states of macrophages.

Furthermore, several histone-modifying enzymes have been reported to participate in the process of macrophage activation and polarization. For example, activation of macrophages can be mediated by histone modifiers such as the SET domain containing 4 (SETD4), an H3K4 methyltransferase [32], whereas SET and MYND domain containing 2 (SMYD2) (H3K36 methyltransferase) and SET and MYND domain containing 5 (SMYD5)(H4K20 methyltransferase) function as negative regulators of macrophage activation [78, 79]. As for macrophage polarization, one epigenetic player is KDM6B, also known as jumonji domain-containing protein-3 (JMJD3), an H3K27-specific demethylase. Increased expression of KDM6B can lead to a reduction of H3K27me3-mediated gene repression at the promoter of M2-related genes, thereby facilitating M2 polarization in anti-helminth host immune responses [80, 81]. Another example is euchromatic histone lysine methyltransferase 2 (EHMT2, also known as G9a), which catalyzes the H3K9me3 modification and represses the expression of the fatty acid transport protein CD36. Over-expression of EHMT2 interrupts fatty acid transport, thereby hindering the process of M1 polarization induced by palmitate, a saturated fatty acid, in favor of M2 polarization [82]. On the other hand, SET and MYND domain containing 3 (SMYD3) is a methyltransferase that catalyzes the active mark and induces expression of S100A9/S100A12, leading to M1 polarization. This epigenetic regulation can be modulated by hyperglycemic conditions [83]. In addition, histone acetylation is frequently involved in M2 polarization [84]. Shinohara et al. showed that colorectal cancer-derived extracellular vesicles promote the M2 phenotype through the downregulation of histone deacetylase 11 (HDAC11) in TAM [38]. Other histone-modifying enzymes that regulate macrophage polarization include SET domain containing 7 (SETD7) for the M1 phenotype, protein arginine methyltransferase 1 (PRMT1) [85] for the M2 phenotype [82], etc., (Table 1).

As chromatin states dictate gene expression patterns, active and repressive chromatin states can co-exist within a nucleus and regulate different sets of genes with diverse functions in mammalian cells. Therefore, the functional effect of a chromatin state is primarily mediated by its target genes. The phenomenon is well exemplified by the fact that multiple histone modifiers with opposite functions work coordinately in the acquisition of either M1 or M2 phenotype in the tumor microenvironment. Understanding the complex interactions among these enzymes and their functional impact may be key to successful therapeutic interventions in the clinic (Fig. 1).

### Epigenetic regulation of the adaptive immune responses—CD8+ T cells

CD8+ T cells are crucial effector cells in adaptive immunity that directly target cancer cells. As a typical response to antigen stimulation during infection or cancer, naïve CD8+ T cells proliferate and differentiate into CD8+ effector T cells that produce anti-tumor effector cytokines. Mounting evidence has indicated that DNA methylation mechanism drives the development of tumor-reactive CD8+ T cells. In colorectal cancer, distinct DNA methylation patterns defined tumor-reactive and bystander CD8+ tumor-infiltrating lymphocytes (TILs). In particular, tumor-reactive markers CD39 and CD103 were specifically demethylated in tumor-reactive CD8+ T cells, along with other signature genes for cytotoxic T cells, such as *PRF1*, *IGNG*, *GZMB*, *CCL3*, *CCL4*, *NKG7*, and *CST7*. Unsurprisingly, the status of DNA demethylation of these immune-related genes demonstrated dynamic changes as naïve T cells develop into tumor-reactive CD8+ T cells[86].

**Fig. 2:**
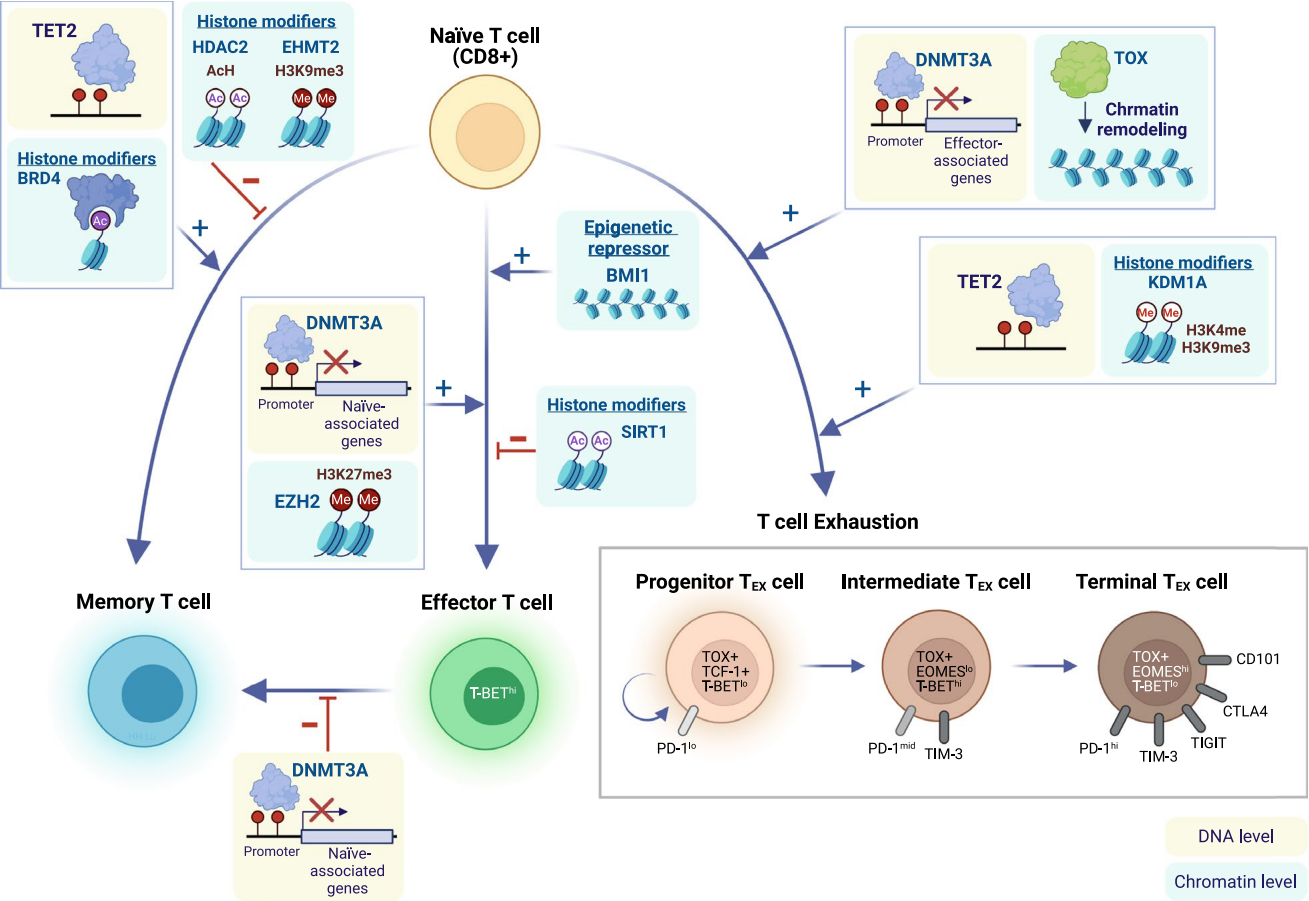
Key epigenetic mediators in the differentiation and exhaustion of CD8+ T cells. Upon antigen stimulation, naïve CD8+ T cells proliferate and differentiate into CD8+ effector T cells that produce anti-tumor effector cytokines. T cells may also adopt a memory phenotype following antigen clearance or an exhausted phenotype after repeated antigen stimulation. Various epigenetic modifiers are involved in the fate determination of naïve CD8+ T cells as they differentiate into effector, memory, or exhausted T (T_EX_) cells. This figure was created on BioRender.com

In addition to DNA methylation, histone modifications and the corresponding histone-modifying proteins also track the differentiation of adaptive immune cells. Upon activation, antigen-specific naïve CD8+ T cells proliferate and differentiate into a heterogeneous pool of effector T cells that consist of two major subsets: short-lived effector cells (SLECs) and memory precursor effector cells (MPECs). Histone modifiers such as EZH2 have been shown to promote SLEC and effector T cell differentiation [53, 87]. Compared with multipotent MPECs that possess developmental plasticity, the pro-memory and pro-survival genes appear to be repressed in SLECs by higher levels of H3K27me3 at gene promoters, leading to fate restriction and limited survival [87]. Consistently, chromatins are more open at genes related to naïve and memory T cell properties in MPECs as opposed to the tightly closed chromatin at these genes in SLECs or exhausted CD8 T cells [88]. In addition, BRD4 has been shown to mediate T cell differentiation into the effector memory phenotype [40], whereas HDAC2 and EHMT2 suppress the development of memory precursor CD8+ T cells [89] (Fig. 2).

EZH2, the histone-modifying enzyme that catalyzes the methylation of histone H3 lysine 27 (H3K27me3), also plays a crucial role in regulating the proliferation and polyfunctionality of effector T cells. Depleting EZH2 in T cells results in poor anti-tumor immunity [53, 90]. By contrast, expression of EZH2 in cancer cells may dampen anti-tumor immunity and hinder the infiltration of the tumor by effector T cells through EZH2- and DNMT1-mediated epigenetic silencing of TH1-type chemokines (i.e., CXCL9 and CXCL10) [91, 92]. Therefore, the regulatory role of histone modifiers in the tumor microenvironment appears to be cell-type-dependent. Striking a delicate balance between anti-tumor and pro-tumor immune responses through pharmacological perturbation of EZH2 is required to achieve therapeutic efficacy.

### Epigenetic regulation of T cell exhaustion

In addition to normal T cell differentiation and activation, epigenetic mechanisms also mediate the dysfunctional state of T cells. Similar to chronic infection, T cells in cancer may experience chronic antigen stimulation and repeated T cell receptor (TCR) signaling that leads to a dysfunctional state, termed exhausted T cells (T_EX_). The exhaustion phenotype is characterized by increased levels of inhibitory receptors (i.e., PD-1, CTLA-4, TIGIT, and TIM-3), defective effector functions, and limited proliferative potential. Notably, T cell exhaustion is not a simple functional switch but a continuous transition process with a stepwise epigenetic reprogramming mediated by different transcription factors. So far, three phases along the trajectory of T cell exhaustion have been described—progenitor, intermediate and terminal T_EX_ cells [3, 8]. Progenitor T_EX_ cells have the ability to proliferate and maintain a plastic dysfunctional state that can be rescued by checkpoint blockade therapy or by removal from the repressive tumor microenvironment [93]. By contrast, terminal T_EX_ cells demonstrate a fixed dysfunctional state that is unresponsive to checkpoint inhibitors and tend to express high levels of CD38, CD101, and EOMES [94, 95].

Evidence has suggested that DNMT3A-mediated de novo DNA methylation program in T cells is required for the acquisition of the exhaustion phenotype. Chronic stimulation of T cells leads to progressive establishment of this exhaustion-associated DNA methylation at genes related to T cell effector responses (i.e., IFN-γ, Myc, Tcf7, and Tbx21), leading to a gradual decline of effector functions. CD8 T cells lacking DNMT3A fail to establish de novo DNA methylation program required for effector-to-exhaustion transition, thereby retaining the effector function despite prolonged antigen stimulation [96]. Notably, DNMTs appeared to be upregulated early in the process of tumor-specific T cells upon tumor antigen encounter and downregulated at the later stage of T cell exhaustion when the dysfunctional state became irreversible [97]. On the other hand, the demethylation process carried out by TET proteins also participates in the trajectory of T cell exhaustion. While DNMTs are essential for initiating the exhaustion program in T cells, TETs are required for the differentiation of progenitor T_EX_ cells completely into terminal T_EX_ cells [98]. Furthermore, TET2-deficient progenitor T_EX_ cells fail to establish satisfactory expansion [98]. These data indicate that, while carrying out opposite molecular functions, both methylation and demethylation enzymes are essential for the process of T cell exhaustion. DNMTs and TETs have distinct protein dynamics and work coordinately to modulate exhaustion-associated DNA methylation programs at different stages of T cell dysfunction.

In addition, T_EX_ cells exhibit a distinct global chromatin accessibility profile from that in naïve, effector, or memory T cells [99]. Interestingly, T_EX_ cells derived from different settings, such as chronic infection or cancer, may share similar epigenetic landscapes [100], indicating that exhausted T cells represent a unique cellular fate rather than an induced response specific to each environmental stimulus. Across various settings of T cell exhaustion, Tox appears to be a master regulator that mediates the development of T_EX_ cell program. Overexpression of Tox can recapitulate key features of exhausted T cells, whereas depletion of Tox partially reverses the T_EX_ phenotype, including reduced chromatin accessibility in inhibitory receptors (i.e., *Pdcd1*) and increased chromatin accessibility near effector cytokine genes (i.e., *GZmA, GZmB*) [101–103]. Chronic TCR stimulation induces activation of NFAT proteins downstream of TCR, leading to Tox expression and changes in chromatin profiles towards the T_EX_ cell phenotype (Fig. 2).

Notably, the T_EX_-associated epigenetic landscape appeared to be relatively stable. Despite the functional reinvigoration of T_EX_ cells by PD-1 blockade, few changes in the intrinsic genome-wide chromatin profiles of T_EX_ cells were observed [104]. The finding may account for the lack of therapeutic durability in many patients receiving immune checkpoint blockade therapy. Similarly, the transfer of T_EX_ cells from mice with chronic LCMV infection into recipient mice without infection failed to convert the exhaustion-associated chromatin state back to the effector/memory state. The stability of exhaustion chromatin patterns represents a persistent “epigenetic scar,” which cannot be easily erased by immune checkpoint blockade or removal from antigen exposure. This epigenetic stability of the exhaustion state is partly maintained by the DNMT3A-mediated de novo DNA methylation program [96]. Genetic disruption or pharmacological inhibition of DNMT3A has been shown to shake the stability of T_EX_ cell state and boost the therapeutic efficacy of PD-1/PD-L1 blockade therapy [96]. Moreover, transcriptomic analysis of tumor-specific T cells in early malignant lesions revealed upregulation of DNA and histone modifying enzymes, including DNMT1, DNMT3B, EZH2, among others, indicating early involvement of the epigenetic machinery in driving the dysfunctional state [97]. In line with these observations, combined treatment with DNMTi and HDACi in a mouse model of non-small cell lung cancer appeared to shift the T cell exhaustion state towards memory and effector T cell phenotypes [105].

### Epigenetic regulation of immunosuppressive cells—Treg

Regulatory T (Treg) cells, immunophenotypically characterized by CD4+CD25+FOXP3+, maintain immune homeostasis and induce self-tolerance. FOXP3 is a potent transcriptional repressor, and its expression correlates with the suppressive capability of Treg. Treg cells may protect hosts from developing autoimmune diseases, but they have detrimental roles in various types of cancers by hindering the development of effective antitumor immunity against tumor cells [106]. The functional and phenotypic heterogeneity of human Treg cells has been reviewed in previous publications [107, 108].

Treg cells possess a number of unique transcriptional and epigenetic features. Upregulated genes in Treg cells, such as Foxp3, Il2ra, Ctla4, Tnfrsf18, and Ikzf2, are located in DNA demethylated regions, which also exhibit open chromatin and the H3K27ac modification [109]. Treg-specific super-enhancer regions, with increased H3K27ac, H3K4me3, and H3K4me1 but decreased H3K27me3, are also associated with Treg signature genes such as *Foxp3*, *Il2ra, Ctla4* and *Ikzf2* [110, 111]. Mounting evidence supports that epigenetic mechanisms determine the plasticity and stability of FOXP3+ T cells. FOXP3 can be acetylated in primary human regulatory T cells, and the enzyme activity of HDAC can destabilize FOXP3+ Treg cells [39]. Another epigenetic modifier, EZH2, is incorporated into FOXP3-containing complexes and deposits repressive chromatin modifications at FOXP3-bound loci [112].

Targeting Treg cells to enhance immune responses against cancer has been an attractive therapeutic approach. Treg cells in tumor tissues specifically express high levels of EZH2, resulting in tumor tolerance. Wang et al. found that disruption of EZH2 activity in Treg cells drives the acquisition of pro-inflammatory functions in Treg cells, enabling the recruitment of effector T cells that eliminate tumors [113]. Similarly, another study by Goswami et al. discovered that the loss of EZH2 function in Treg cells can reprogram their phenotype to effector-like T cells and induce robust antitumor immunity. Notably, pharmacological inhibition of EZH2 expression enhances the effectiveness of anti-CTLA-4 therapy [54]. Thus, targeting Treg cells, alone or in combination with other anti-cancer treatments, holds promises in providing therapeutic benefits for cancer patients.

### Epigenetic regulation of antigen presentation cells—dendritic cells

Dendritic cells (DCs) are highly specialized antigen presenting cells that serve to activate T cells by presenting tumor antigens. Conventionally, DCs were classified into three groups: conventional DCs (cDCs), plasmacytoid DCs (pDCs), and monocyte-derived DCs (mDCs) [114]. Depending on different types of inflammatory stimuli, DCs may be specifically tailored to produce different sets of cytokines and induce heterogeneous T cell responses [115]. Traditionally only cDCs were thought to be critical in anti-tumor response. Yet, new evidence suggested that other DCs, such as mDCs, were also essential in the favorable immune response to tumors [116]. Murine models suggested that specific transcriptional factors (TFs), such as SPI1 (also known as PU.1), and STAT3, control the development of DC from progenitors in the bone marrow [117–120]. Further studies revealed that epigenetic mechanisms, in conjunction with transcriptional factors, can influence the maturation of DCs. An integrative analysis of epigenetic modifications and transcriptional factor occupancy revealed that DC-specific epigenetic signature and PU.1 occupancy increasingly colocalized during DC commitment and specification [121].

Among all key epigenetic players involved in DC maturation, one group of enzymes are HDACs. A study demonstrated that the maturation of DCs from monocyte was blunted by HDAC inhibitors, which was associated with obstructed signaling through nuclear TFs, including nuclear factor-κB, IRF-3, and IRF-8 [122]. HDAC inhibitors were also shown to inhibit the differentiation of both mDCs and pDCs via attenuating the expression of PU.1 [123]. EZH2, an epigenetic regulator, was also crucial in the maturation of both mDCs and pDCs; treatment with an EZH2 inhibitor may suppress DC-driven T cell proliferation [124]. In addition, DOT1L, a H3K79 methyltransferase, has been shown to play a suppressive role in DC maturation. Inhibition of DOT1L reduced H3K79me2 enrichment at the FOXM1 promoter, thereby downregulating FOXM1 and promoting IL-12 production and DC maturation in colon and pancreatic cancers [31].

Epigenetic mechanisms not only mediate DC maturation but also govern the recruitment of pDCs from bone marrow to the tumor microenvironment. Nicotinamide adenine dinucleotide (NAD)^+^-dependent deacetylase SIRT6 acts on histone H3K9 acetylation and promotes CXCR4+ DCs’ migration to lymph nodes in an animal model of multiple sclerosis [36]. Moreover, in distinct lineages of lung dendritic cells, mDCs showed lower migration and low expression of CCR7 compared with cDCs. It was found that different levels of H3K27me3 at *CCR7* gene were observed between migratory cDCs and non-migratory mDCs [125]. These data suggest that epigenetic framework can influence the maturation and recruitment of DCs, thereby modulating the interactions between DCs and other immune cells in the tumor microenvironment.

### Epigenetic regulation of immunosuppressive cells—MDSC

Myeloid-derived suppressor cells (MDSCs) are immature myeloid cells that have immunosuppressive activities in TIME. MDSCs are mainly divided into three phenotypically distinct subpopulations: CD33 + HLA-DR^−/low^CD14 − CD15 − immature MDSCs (I-MDSC), CD33 + HLA-DR^−/low^ CD14 + CD15 − monocytic MDSCs (M-MDSCs), and CD33 + HLA-DR^−/low^CD14 − CD15 + granulocytic MDSCs (G-MDSCs or PMN-MDSCs) [126]. The differentiation and functional states of MDSC are at least, in part, regulated by epigenetic mechanisms. Transcriptomic analysis revealed differential expression of genes involved in DNA methylation and histone modifications between different tumor-infiltrating MDSC subsets. Genes related to HDAC activation and DNA methylation were significantly upregulated, while genes related to HAT activity were downregulated in tumor-infiltrating I-MDSCs. By contrast, genes related to HDAC activation and DNA methylation were downregulated in tumor-infiltrating G-MDSCs [127]. Notably, different classes of HDAC may exert distinct effects on MDSCs. For instance, HDAC11 (Class VI) negatively regulates MDSC expansion and immunosuppressive function [128]; HDAC2 (Class I) facilitates a phenotypic switch from M-MDSC to G-MDSC [37].

In line with the above observations, HDAC inhibitors may selectively target different MDSC subsets. Hashimoto et al. demonstrated that treatment with entinostat, a class I HDAC inhibitor, reduced the immune suppressive activity of only G-MDSC, whereas it had no effect on M-MDSC, which had a higher amount of HDAC6 (class IIb HDAC). Inhibition of HDAC6 with ricolinostat reduced the suppressive activity of M-MDSC but not G-MDSC. A combination of entinostat and ricolinostat was needed to inhibit both populations of MDSCs and hinder tumor progression [129] (Table 1). The suppressive effects of epigenetic modulating agents on MDSCs were demonstrated in another study where suppression of MDSC by combined treatment of azacitidine (DNMTi) and entinostat (HDACi) could sensitize metastatic mouse cancers to immune checkpoint blockade (anti-PD-1 and CTLA-4) or inhibit metastases by disrupting premetastatic niches established by MDSCs [130, 131].

## Epigenetic regulation of the tumor-immune interface: immune synapse

In addition to the individual immune cell types mediated by epigenetic programs, epigenetic modulation of the tumor-immune interface can also be a potential therapeutic strategy. Immunological synapse refers to a specialized structure formed at the interface between a T cell and a cancer cell or a T cell and an antigen presenting cell (APC), which is a key event mediating T cell activation in adaptive immune responses [132, 133]. The assembly of a classical lytic immune synapse starts with a TCR recognizing a cognate MHC molecule loaded with a specific antigenic peptide [133]. The interaction triggers a series of events, including clustering of co-stimulatory receptors, adhesion molecules, and cytoskeletal elements, leading to the activation of multiple signaling pathways and expression of effector molecules. The lytic immune synapse has three major structure compartments—central, peripheral and distal supramolecular activation clusters (cSMAC, pSMAC, and dSMAC) [134]. cSMAC is composed of TCR and associated signaling molecules, including protein kinase C (PKC)-θ and Lck; pSMAC contains integrin LFA-1, talin, and other adhesion molecules; dSMAC primarily includes the accumulation of actin cytoskeleton. Together, the three ring-like compartments create a “bull-eye” configuration [135]. In the case of cytotoxic CD8+ T cell-target cell encounters, the formation of the immune synapse triggers polarization of the microtubule-organizing center (MTOC) towards cSMAC, which facilitates directional and coordinated delivery of lytic granules (i.e., perforin and granzymes) into cancer cells, leading to specific killing. Similarly, immune synapse formation can also be found at the interface between cancer cells and other specialized cytotoxic cells such as natural killer cells, γδ T cells, and even engineered chimeric antigen receptor (CAR) T cells. The formation of a functionally robust immune synapse is essential for cytotoxic immune cell-mediated cancer killing.

Emerging evidence has suggested that the sophisticated process of immune synapse formation and the resulting lytic function can be regulated by epigenetic processes [136, 137]. In fact, many immune synaptic molecules are regulated by DNA methylation. Berglund et al. analyzed DNA methylation data of immune synaptic genes in 30 solid tumor types from the Cancer Genome Atlas (TCGA) database and found that many costimulatory genes are hypermethylated and inhibitory immune checkpoint genes are hypomethylated in cancer [137]. Consistently, treating cancer cells with DNMT inhibitors can demethylate and upregulate MHC genes [138, 139]. By contrast, cancer cells express inhibitory synaptic proteins such as immune checkpoints via DNA demethylation to suppress the immune system. Collectively, these DNA methylation-mediated events play a significant role in cancer immune evasion. Thus, DNA methylation patterns of immune synapses reflect the immunogenicity of cancer cells.

**Fig. 3:**
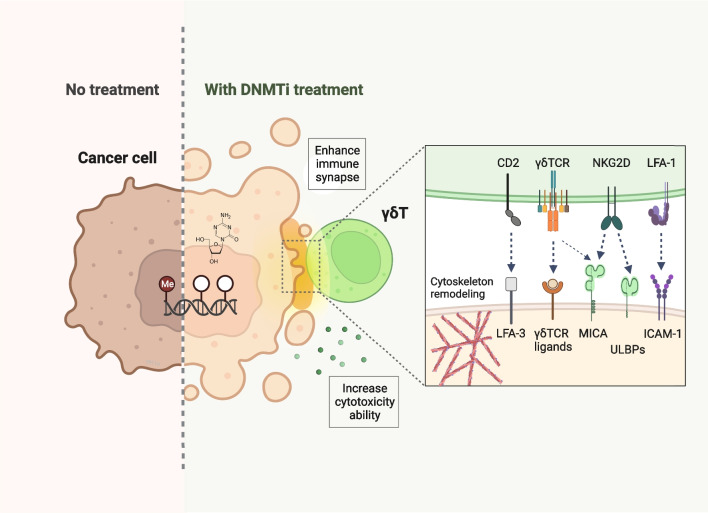
Epigenetic therapy enhances immune synapse formation between cancer and γδ T cells. Epigenetic therapeutic agents, such as DNA methyltransferase inhibitors (DNMTis), upregulate the expressions of multiple immune synaptic molecules, such as ICAM-1, MICA, and ULBPs on cancer cells, and NKG2D on γδ T cells. The drugs significantly facilitate immune synapse formation between cancer and γδ T cells, and potentiate γδ T-mediated tumor lysis. This figure was created on BioRender.com

In addition, the assembly or quality of immune synapses has high clinical relevance and can serve as a therapeutic target in the treatment of cancer. Our group previously showed that pharmacological depletion of DNMTs in lung cancer cells led to upregulation of multiple immune synaptic molecules by quantitative surface proteomics analysis, including MHC, stimulatory or inhibitory immune checkpoints, adhesion molecules, as well as ligands for MHC-unrestricted receptors of innate immune cells. Remarkably, treating cancer cells with DNMTi significantly enhanced immune synapse formation and cytoskeletal reorganization, leading to stronger killing of cancer cells by cytotoxic γδ T cells [136] (Fig. 3). As more and more epigenetic mechanisms underlying the functional switch and architectural reorganization of various synaptic molecules are being discovered, it is anticipated that fine-tuning of immune synaptic assembly by epigenetic drugs may hold great potential for developing therapeutic strategies in the management of cancer.

## Epigenetic therapy and TIME modulation

Owing to the well-established role of epigenetic dysregulation in the origin and progression of cancer, many efforts have been invested in the development of epigenetic drugs for the treatment of cancer. Currently, several agents targeting three epigenetic enzymes—DNMT, HDAC, and EZH2—have been approved by the U.S. FDA for treating diverse malignancies. For instance, DNA methyltransferase inhibitors (i.e., azacitidine, decitabine, onureg, and inqovi) were approved for the treatment of myelodysplastic syndromes (MDS)/acute myeloid leukemia (AML). An EZH2 inhibitor (i.e., tazemetostat) was approved for follicular lymphoma (FL). Three HDAC inhibitors (i.e., romidepsin, belinostat, vorinostat) were approved for cutaneous T-cell lymphoma (CTCL), and another HDAC inhibitor, panobinostat, was approved for treating multiple myeloma. Meanwhile, a wide range of epigenetic-based drugs are undergoing clinical trials. It is increasingly appreciated that pharmacological inhibition of epigenetic enzymes not only can have therapeutic benefits against malignant cells but also facilitate anti-tumor immunity via modulation of the TIME.

### DNA methyltransferase inhibitors (DNMTis)

Azacitidine and decitabine are cytidine analogues that incorporate into DNA or RNA, and inhibit the activity of DNMTs. These drugs have been shown to produce an antitumor “memory” response, accompanied by sustained decreases in genome-wide promoter DNA methylation, gene re-expression, and alterations in major cancer-intrinsic signaling pathways [138]. On the other hand, abundant evidence has indicated that DNMTis possess immunomodulatory functions that may constitute part of their anti-tumor effect. In a variety of cancer types, DNMTi may augment cancer cell immunogenicity through re-expression of tumor-associated antigens (e.g., cancer testes antigens), antigen processing, and antigen presentation machinery (e.g., MHCs) [139, 140]. The immune-modulatory effects of DNMTi have further been linked to activation of endogenous retroviral sequences and induction of anti-viral interferon responses, which enhances tumor cells’ response to anti-CTLA-4 checkpoint blockade therapy [141, 142]. At the same time, upregulation of the immune checkpoint molecules (e.g., PD-L1, PD-L2, etc.) by DNMTis may also contribute to the enhancing effects of checkpoint blockade therapy [140]. On the other hand, this modulatory effect on immune checkpoints can be a double-edged sword. In MDS and AML patients treated with DNA hypomethylating agents alone, upregulation of these inhibitory immune checkpoint molecules has been associated with therapeutic resistance [143].

**Fig. 4:**
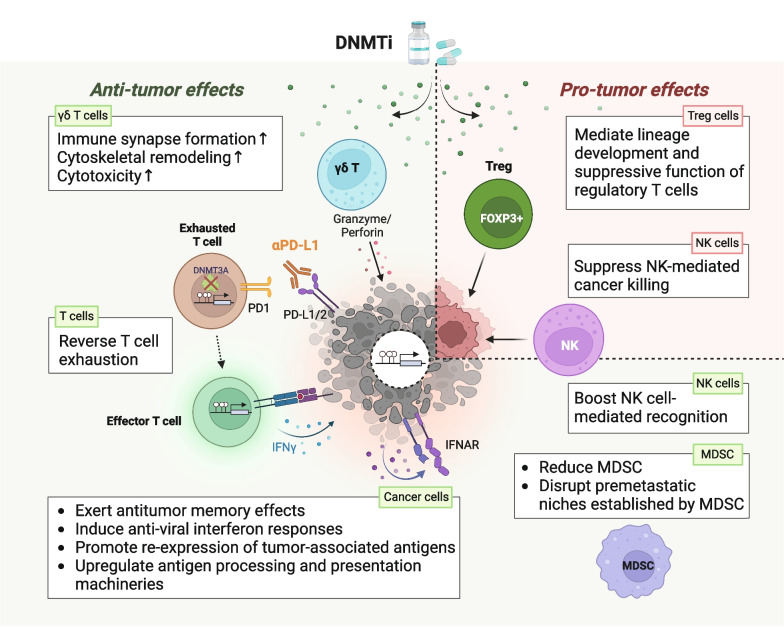
Therapeutic effects of DNMT inhibitors on different types of immune cells in the tumor immune microenvironment. DNA methyltransferase inhibitors (DNMTis) possess both anti-tumor and pro-tumor effects via reprogramming different immune cell types in TIME. DNMTi treatment of cancer cells exerts anti-tumor memory effects, induces anti-viral interferon responses, promote expression of tumor-associated antigens, as well as upregulation of antigen processing and presentation machineries, which enhances the recognition and lysis of cancer cells by effector T cells. Besides, DNMTi appears to reinvigorate dysfunctional T cells and shift the exhaustion state towards an effector T cell phenotype. Furthermore, DNMTi significantly promotes immune synapse formation and cytoskeletal reorganization, leading to robust cancer killing by cytotoxic γδ T cells. Besides, DNMTi promotes anti-tumor immunity by reducing myeloid-derived suppressor cells (MDSC) and disrupting premetastatic niches established by MDSC. Interestingly, DNMTis show bifacial effects on natural killer (NK) cells and may either suppress NK cell-mediated cancer killing or boost NK cell-mediated recognition of cancer cells. On the other hand, DNMTi may exert pro-tumor effects by promoting lineage development and suppressive function of Treg. This figure was created on BioRender.com

The immunomodulatory effects of DNMTi on immune cells have also been described. In patients with advanced solid tumors, low-dose decitabine treatment promoted activation and proliferation of IFN-γ^+^ T cells to enhance antitumor immunity and improve clinical responses [144]. In addition, DNMTi has been found to reverse T cell exhaustion [105] or remove the methylation barrier to T cell rejuvenation mediated by immune checkpoint blockade [96], consistent with the findings that the T cell exhaustion state is mediated by DNMT3A-mediated de novo DNA methylation program [96]. Interestingly, DNMTi appears to fine-tune the immune system in a sophisticated way. In 27 AML patients undergoing allogeneic stem cell transplantation, treatment with azacitidine augmented a graft-versus-leukemia (GVL) effect by inducing cytotoxic CD8+ T-cell responses to tumor antigens on leukemic blasts. At the same time, azacitidine maintains a delicate immunological balance through increasing Treg to avoid graft-versus-host disease (GVHD) [145, 146]. Intriguingly, the DNMTi’s immunomodulatory effects are immensely pleiotropic. As shown in Fig. 4, the differential effects of DNMTi on individual immune cells in TIME can exert either anti-tumor or pro-tumor responses. Therefore, it can be clinically challenging to evaluate the overall impact of DNMTi on positive and negative immune regulators and to predict clinical outcomes of cancer patients. This facilitates multiple efforts toward identifying markers in TIME as the determinants of clinical response to DNMTi. For instance, increased IFN-γ-producing cells and increased T-cell cytotoxicity following decitabine treatment predicted improved therapeutic responses and survival in clinical patients [144]. In addition, AML patients with elevated pretreatment T cell diversity and those with an increased T cell receptor beta repertoire richness after azacitidine treatment had longer event-free and overall survival [147]. These data highlight the importance of TIME in dictating the effects of DNMTi on MDS/AML.

### EZH2 inhibitors

EZH2 is a subunit of the polycomb repressive complex 2 (PRC2) complex that regulates gene expression by catalyzing trimethylation of histone H3 lysine 27. The enzymatic inhibition of EZH2 has been intensively studied as a therapeutic strategy in cancer. Two leading compounds, EPZ-6438 and GSK126, have shown encouraging clinical benefits in diffuse large B cell lymphoma (DLBCL) and FL [148]. EPZ-6438, also known as tazemetostat, has been approved by the U.S. FDA to treat FL. The major effect of EZH2i works on cancer cells with EZH2-activating mutations. However, clinical studies have shown that it is also effective for FL without EZH2 mutations [149]. Since EZH2 is a key epigenetic mediator in many immune cell types as mentioned above, emerging data also reveal the critical role of EZH2i in TIME remodeling. Tazemetostat upregulates the expression of CCL17 in B-cell lymphoma and enhances the recruitment of IFN-γ secreting T cells. In human lymphoma databases, the CCL17 expression level is inversely correlated with the EZH2 activation gene signature and is significantly associated with T-cell–rich microenvironment in FL and Germinal Center B-Cell like (GCB) DLBCLs [150]. In addition, EZH2 inhibition resulted in significant upregulation of MHC class I expression in mouse models in vivo. Enhanced antigen presentation on the tumor cells by EZH2 inhibitors or CRISPR-mediated EZH2 deficiency have been shown to increase antigen-specific CD8+ T-cell proliferation, IFN-γ production, and tumor cell cytotoxicity [151].

### Histone deacetylase inhibitors (HDACis)

Vorinostat, panobinostat, and belinostat are pan-HDAC inhibitors and act on class I, II, and IV of histone deacetylases. Romidepsin primarily functions by inhibiting class I HDACs with only weak effects on the class IIb HDAC6. HDACis could modulate the TIME indirectly via the communication of tumor cells and immune cells. For instance, romidepsin increases MICA/B on AML cells, which in turn promotes antibody-dependent phagocytosis of AML cells by macrophages [152]. Romidepsin also induces NKG2D ligand expression on lung cancer cell lines and improves NK cell-mediated anti-cancer immunity [153]. Vorinostat upregulates OX40L in Hodgkin lymphoma cells. Engagement of OX40L with OX40 receptor on Treg cells and NK cells alters the antitumor immunity [154].

Abundant evidence has revealed direct effects of HDACis on immune cells in TIME. Similar to DNMTi, HDACis’ immunomodulatory effects are complex. HDACis alter the cytokines in favor of a TH1-type immune response. HDAC inhibitors have been shown to reduce the frequencies of regulatory T cells and MDSCs [155]. Moreover, HDACi’s rewiring of pDCs enhances anti-tumor immunity via activation of IFN-γ and TNF pathways. Depletion of pDCs abrogates panobinostat-mediated induction of type I IFN signaling in AML cells and impairs therapeutic efficacy [156]. In addition, panobinostat can trigger TNF secretion in lymphocytes and enhance lymphocyte-mediated lysis of Hodgkin lymphoma cells [157]. In multiple myeloma, Hodgkin lymphoma, and other solid tumors, panobinostat or ACY241(an HDAC6 selective inhibitor) have demonstrated suppressive effects on the expression of the inhibitory checkpoint PD-1 in CD8+ T cells, which may further enhance their anti-tumor immunity [155, 158]. Interestingly, different HDACis may have opposite modulatory effects on the expressions of immune checkpoints. Despite that several HDACis appear to downregulate PD-1 expression, VA may upregulate PD-1 expression in CD8+ T cells from AML patients [159].

### Bromodomain and extra-terminal motif (BET) protein inhibitors

In addition to histone-modifying enzymes that add or remove histone modifications, targeting epigenetic readers that recognize specific functional groups on histone tails may also confer regulatory or therapeutic activities in the TIME. Bromodomain and extra-terminal domain (BET) proteins—recognize acetylated lysine of histones [160] or acetylated nonhistone transcription factors [161]. BET proteins are crucial in gene expression regulation and act as a scaffold to form a complex with other proteins at gene promoters or enhancers to facilitate gene activation[160, 162]. Depletion of BRD2 or BRD4, members of the BET protein family, is lethal for embryonic development [163, 164]. In addition, BET proteins play an important role in cancer development. BRD2 and BRD4 are overexpressed in human cancer cells [165, 166]. The most well-known involvement of BET proteins in cancer is from NUT (nuclear protein in testis) carcinoma, a rare form of undifferentiated carcinoma involving mid-line structures. NUT carcinoma is characterized by the fusion of the NUT gene with BET genes such as *BRD4* or *BRD3* [167]. Silencing of BRD-NUT proteins resulted in squamous cell differentiation and cell cycle arrest [167]. BET inhibitors (BETi), such as JQ-1 and OTX015, have been shown to have anti-tumor activities in NUT carcinoma in preclinical studies and clinical trials [168, 169]. Nevertheless, clinical trials for the anti-tumor effect of OTX015 on other solid tumors showed mixed results. To date, BET protein inhibitors have not yet shown positive results in phase 3 trials.

Furthermore, BET proteins in cancer can regulate TIME through activating proinflammatory genes. For instance, BRD4 was required for IL-6-stimulated and Notch1-induced cancer migration in a triple-negative breast cancer model, indicating that BRD4 linked microenvironment inflammation to cancer propagation [170]. Dual inhibition of BRD4 and PI3K repressed IL-4-driven macrophages and their immunosuppression effect in syngeneic and spontaneous murine cancer models [171]. Moreover, treatment with a bromodomain inhibitor, I-BET 762, reduced the number of macrophages in pancreatic and lung cancer animal models [172, 173]. BRD4 not only regulates innate immune cells but also modulates the adaptive immunity. JQ1, a BET inhibitor, increased the frequency of dendritic cells and CD8+ T cells and reduced the expansion of MDSC in a malignant pleural mesothelioma murine model [174]. This immune-modulatory function of BETi can be attributed to extrinsic regulation from BETi-treated cancer cells as well as intrinsic regulation from BETi-treated immune cells. BETis have been shown to transcriptionally downregulate inhibitory immune checkpoints [175, 176], rescue PD-1-mediated T cell exhaustion [177], and enhance T cell persistence in adoptive immunotherapy [40]. On the other hand, BET inhibition may also be associated with impaired T cell proliferation [178] and reduced IFN-γ production in multiple immune cell types [179]. Interestingly, despite the mixed immunomodulatory effects of BET inhibition on various immune cells, combined treatment with BETi and checkpoint inhibitors has shown therapeutic benefits in various preclinical models [180, 181]. These findings highlight the need for a further in-depth investigation on the complex and dynamic effects of BET inhibition in the TIME. It is possible that the effect of BETi is dose-dependent or context-dependent. Inhibiting BET proteins at different stages of T cell activation and differentiation or in a “hot” or “cold” immune background may lead to different outcomes.

## Conclusions and future perspective

Major epigenetic mechanisms—DNA methylation, histone modifications, and chromatin remodeling—collectively govern the functional state transitions of various immune cells in the TIME. Preclinical and clinical studies on the combination of epigenetic drugs and various immunotherapies have supported the idea of remodeling TIME as promising therapeutic strategies [1, 96, 182, 183]. In addition to the endogenous immune cells, epigenetic therapy has been shown to modulate adoptively transferred effector cells. HDAC inhibitors such as panobinostat can promote a central memory phenotype of dual-specific CAR T cells [184]. DNMT inhibitors such as decitabine may enhance the polyfunctionality of ex vivo expanded γδ T cells [136]. Nevertheless, more questions remain to be answered.

First, our understanding of these epigenetic regulatory processes was mostly based on studies focusing on a single immune cell type or research findings derived from pooled cell populations. Analysis of the bulk tumor transcriptomic data using techniques such as CIBERSORT [185] and XCell [186] has enabled us to computationally visualize cellular components within the TIME. Still, the accuracy of data output relies heavily on the availability, quality, and completeness of a reference gene matrix containing the immune cell subtypes of interest. With a variety of single-cell and spatial technologies (e.g., single-cell RNA-seq, single-cell ChIP-seq, single-cell ATAC-seq, spatial transcriptomics, mass cytometry and etc.) being developed [187–192], researchers can now look into the sophisticated interactions among cellular components within the TIME and how its constituents collectively affect the therapeutic efficacy in primary tumor tissues. Furthermore, cancer patients receiving clinical epigenetic therapy can provide an ideal research platform for characterizing epigenetic regulatory effects on the TIME in vivo, where the cancer-immune interaction is intact.

Second, regulatory effects of individual epigenetic modifiers can be cell-type specific and sometimes lead to opposite functional outcomes. On the same token, epigenetic therapy may have conflicting effects on different immune cell types. For example, DNMT inhibitors appear to promote anti-tumor immunity by enhancing effector function of T cells and reversing the exhausted state of T cells. On the other hand, the drugs have been shown to impede NK-mediated cancer killing or increase the number of immunosuppressive Treg cells in some cases. How to strike a fine balance between positive and negative immunomodulatory effects to achieve optimal anti-tumor immune responses can be clinically challenging. Moreover, the TIME resembles a dynamic ecosystem that can be altered along the trajectory of tumor development or following various types of therapy. The heterogenic nature of the TIME is further complicated by the fact that metastatic loci may or may not share common immune characteristics with primary tumors, even within the same patients. Thus, efforts to identify key determinants that drive the beneficial epigenetic immunomodulatory effect will be needed for implementing precision immunotherapy.

Furthermore, epigenetic regulation of the TIME is often complicated by epigenetic plasticity and immune cell plasticity, which can constitute a barrier to effective cancer treatment. The chromatin state is an essential determinant of cellular phenotype. Notably, aberrant restrictive or permissive chromatin is considered a hallmark of cancer [193]; thus, both increased and decreased epigenetic plasticity can be observed in cancer. Increased epigenetic plasticity allows cancer cells to adopt a new transcription program and metabolic state. One example is the loss of CTCF insulator and subsequent activation of oncogenes. By contrast, decreased epigenetic plasticity, also known as epigenetic restriction, results from promoter DNA hypermethylation or excess polycomb-mediated transcriptional repression. Decreased epigenetic plasticity renders the cells unable to activate differentiation or self-renewal programs. One of the best examples comes from gain-of-function mutations of EZH2 in lymphoma and melanoma. Increased H3K27me3 by EZH2 blocks B cell differentiation and arrests these B cells in a proliferative state [194]. Likewise, immune cell plasticity may be a general phenomenon rather than a rare exception in TIME. T cell plasticity toward either effector or exhausted phenotype is subject to epigenetic regulation. Macrophage plasticity toward M1 or M2 polarization is also epigenetically controlled. Notably, the intertalks between epigenetically-mediated plasticity in different immune cell types further add complexity to the entire ecosystem of the TIME. Therefore, understanding the interaction between epigenetics and the immune system in each cancer subtype remains a challenge.

Lastly, as more and more epigenetic therapeutic agents are entering clinical trials or in the process of seeking government approval, identification of epigenetic targets that mediate the functional attributes of the TIME may enhance the repertoire of immunotherapeutic strategies in the clinic. Emerging epigenetic targets, such as BRD4 [33], KDM1A [33], NR4A1 [195], have shown promising activities in modulating T cell function or persistence. A recent study by Belk et al. utilized a series of in vitro and in vivo CRISPR-Cas9 screens and identified ARID1A as a novel epigenetic remodeling factor mediating terminal exhaustion of T cells [196]. While more preclinical or clinical investigations will be needed to demonstrate real therapeutic benefits, these studies open exciting new possibilities to facilitate new drug development or repurposing of existing drugs. As we gain deeper insights into the regulatory networks driving immune-immune and immune-tumor interactions, physicians and scientists will be empowered to develop novel therapeutic strategies to convert the tumor-permissive TIME to a functionally-inflamed immune landscape that promotes effective and durable clinical benefits.

## Data Availability

Not applicable.

## Abbreviations

5hmC: 5-Hydroxymethylcytosine
AML: Acute myeloid leukemia
APC: Antigen presenting cell
ARID1A: AT-rich interaction domain 1A
ASH1L: ASH1 like histone lysine methyltransferase
ATAC-seq: Assay for transposase-accessible chromatin using sequencing
BRD4: Bromodomain-containing protein 4
CAR-T: Chimeric antigen receptor T cell
CCL17: C–C Motif chemokine ligand 17, also known as TARC
CCL3: C–C Motif chemokine ligand 3
CCL4: C–C Motif chemokine ligand 4
CCR7: C–C Motif chemokine receptor 7
CD101: CD101 molecule
CD38: Cluster of differentiation 38
cDCs: Conventional dendritic cells
ChIP-seq: Chromatin immunoprecipitation (ChIP) sequencing
CRISPR: Clustered regularly interspaced short palindromic repeats
cSMAC: Central supramolecular activation clusters
CST7: Cystatin F
CTCL: Cutaneous T-cell lymphoma
CTLA-4: Cytotoxic T-lymphocyte associated protein 4
CXCL10: C-X-C Motif chemokine ligand 10
CXCL9: C-X-C Motif chemokine ligand 9
CXCR4: C-X-C Motif chemokine receptor 4
DCs: Dendritic cells
DLBCL: Diffuse large B cell lymphoma
DNAM1: DNAX accessory molecule-1, also known as CD226 (cluster of differentiation 226)
DNMT: DNA methyltransferase
DOT1L: DOT1 like histone lysine methyltransferase
dSMAC: Distal supramolecular activation clusters
EHMT2: Euchromatic histone lysine methyltransferase 2
EOMES: Eomesodermin
EZH2: Enhancer of zeste homolog 2
FOXM1: Forkhead Box M1
FOXP3: Forkhead Box P3
G-MDSCs: Granulocytic myeloid-derived suppressor cell
GCB: Germinal center B-cell like
GVHD: Graft-versus-host disease
GVL: Graft-versus-leukemia
GZMA: Granzyme A
GZMB: Granzyme B
HAVCR2: Hepatitis A virus cellular receptor 2
HATs: Histone acetyltransferases
HDACs: Histone deacetylases
HDAC11: Histone deacetylase 11
HDAC6: Histone deacetylase 6
HLA-DR: Human leukocyte antigen–DR isotype
HMTs: Histone methyltransferases
I-MDSC: Immature myeloid-derived suppressor cell
IFN-γ: Interferon gamma
IKZF2: IKAROS family zinc finger 2
IL-1: Interleukin 1
IL-10: Interleukin 10
IL-12: Interleukin 12
IL-15Rβ (CD122): Interleukin 15 receptor, beta, also known as IL2RB (interleukin 2 receptor subunit beta)
IL-4: Interleukin 4
IL-6: Interleukin 6
INO80: INO80 complex ATPase subunit
IRF-3: Interferon regulatory factor 3
IRF-8: Interferon regulatory factor 8
ISWI: Imitation switch chromatin remodeling complex
JARID2: Jumonji and AT-rich interaction domain containing 2
JMJD3: Jumonji domain-containing protein-3, also known as KDM6B (lysine demethylase 6B)
KDM: Lysine demethylase
KDM1A: Lysine-specific histone demethylase 1A, also known as LSD1
KDM6B: Lysine demethylase 6B, also known as JMJD3 (jumonji domain-containing protein-3)
KIR2DL1-3: Killer cell immunoglobulin like receptor, two Ig domains and long cytoplasmic tail 1–3
KIRs: Killer Ig-like receptors
Lck: LCK proto-oncogene, Src family tyrosine kinase
LCMV: Lymphocytic choriomeningitis virus
LFA-1: Lymphocyte function-associated antigen 1, also known as ITGAL (integrin subunit alpha L)
M-MDSC: Monocytic myeloid-derived suppressor cell
mDCs: Monocyte-derived dendritic cells
MDS: Myelodysplastic syndromes
MDSC: Myeloid-derived suppressor cell
MHC: Major histocompatibility complex
Mi-2/NuRD: Nucleosome remodeling and deacetylase complex
MICA: MHC class I polypeptide-related sequence A
MICB: MHC class I polypeptide-related sequence B
MPECs: Memory precursor effector cells
MTOC: Microtubule-organizing center
NAD: Nicotinamide adenine dinucleotide
NCR1: Natural cytotoxicity triggering receptor 1, also known as NKp46 or CD335 (cluster of differentiation 335)
NFAT: Nuclear factor of activated T-cells
NK: Natural killer cells
NKG2A: Natural killer group 2A, also known as KLRC1 (killer cell lectin like receptor C1)
NKG2D: Natural killer group 2D, also known as KLRK1 (killer cell lectin like receptor K1)
NKG7: Natural killer cell granule protein 7
NKp30: Natural killer protein 30, also known as CD337 (cluster of differentiation 337)
NR4A1: Nuclear receptor subfamily 4, group A, member 1
OX40: Also known as TNFRSF4 (TNF receptor superfamily member 4)
OX40L: Also known as TNFSF4 (TNF superfamily member 4)
PD-1: Programmed cell death protein 1
pDCs: Plasmacytoid dendritic cells
PDCD1: Programmed cell death protein 1
PKC: Protein kinase C
PMN-MDSC: Polymorphonuclear myeloid-derived suppressor cell
PRF1: Perforin 1
PRMT1: Protein arginine methyltransferase 1
pSMAC: Peripheral supramolecular activation clusters
SAM: S-adenosylmethionine
SETD4: SET domain containing 4
SETD7: SET domain containing 7
SIRT6: Sirtuin 6
SLECs: Short lived effector cells
SMYD2: SET and MYND domain containing 2
SMYD5: SET and MYND domain containing 5
SPI1: Spi-1 Proto-oncogene, also known as PU.1
STAT3: Signal transducer and activator of transcription 3
SWI/SNF: SWItch/sucrose non-fermentable complex
TAM: Tumor-associated macrophage
TCGA: The Cancer Genome Atlas
TCR: T cell receptor
TET: Ten-eleven translocation enzyme
TFs: Transcriptional factors
TGF-β: Transforming growth factor beta
TH1: T helper type 1
TIGIT: T cell immunoreceptor with Ig and ITIM domains
TIM-3: T-cell immunoglobulin and mucin-domain containing-3 also known as HAVCR2
TIME: Tumor immune microenvironment
TNF: Tumor necrosis factor
TNFRSF18: TNF receptor superfamily member 18
TRAIL: Tumor necrosis factor-related apoptosis-inducing ligand, also known as TNFSF10 (TNF superfamily member 10)
Treg: Regulatory T cells
TSA: Trichostatin A
U.S. FDA: United States Food and Drug Administration
ULBPs 1–6: UL16 binding protein 1–6
UTY: Ubiquitously transcribed tetratricopeptide repeat containing, Y-linked
VA: Valproic acid
